# Stiffness of the Gastrocnemius–Achilles Tendon Complex Between Amateur Basketball Players and the Non-athletic General Population

**DOI:** 10.3389/fphys.2020.606706

**Published:** 2020-12-10

**Authors:** Tian-Tian Chang, Zhe Li, Xue-Qiang Wang, Zhi-Jie Zhang

**Affiliations:** ^1^Department of Sport Rehabilitation, Shanghai University of Sport, Shanghai, China; ^2^The First Clinical Medical School, Shaanxi University of Chinese Medicine, Xi’an, China; ^3^Rehabilitation Therapy Center, Luoyang Orthopedic Hospital of Henan Province, Orthopedic Hospital of Henan Province, Luoyang, China

**Keywords:** stiffness, adaptation, Achilles tendon, gastrocnemius, basketball

## Abstract

Muscle and tendon stiffness are related to sports performance, tendinopathy, and tendon degeneration. However, the effects of habitual loading on muscle and tendon mechanical properties are unclear. Using amateur basketball players as examples, we investigated the effects of mechanical loading on the stiffness of the gastrocnemius–Achilles tendon (AT) complex in non-dominant and dominant lower limbs. Then, we evaluated the correlation between gastrocnemius and AT stiffness. Forty participants (20 amateur basketball players; 20 normal non-athletic persons) were recruited for this study. Stiffness of the gastrocnemius–AT complex was assessed using MyotonPRO at neutral position and 10° dorsiflexion of the ankle joint in participants from amateur basketball players and the non-athletic general population. Our results showed a greater stiffness of the gastrocnemius–AT complex in amateur basketball players than that in healthy non-athletic subjects at neutral position and 10° dorsiflexion of the ankle joint (*P* < 0.05). No significant difference in stiffness was found between the non-dominant and dominant lower limbs either in amateur basketball players or in generally healthy subjects (*P* > 0.05). A significant positive correlation was obtained between stiffness of the AT and medial gastrocnemius (MG) in amateur basketball players (neutral position: *r* = 0.726 and *P* = 0.001; dorsiflexion 10°: *r* = 0.687 and *P* = 0.001). The amateur basketball players exhibit significantly higher stiffness value in Achilles and gastrocnemius. This is possibly caused by repeated training effects. The symmetric stiffness of the AT and gastrocnemius exists both in amateur basketball players and generally healthy subjects. A significant correlation between the AT and the MG was found in amateur basketball players.

## Introduction

The gastrocnemius–Achilles tendon (AT) complex, consisting of the medial gastrocnemius (MG), and lateral gastrocnemius (LG) and AT, is the largest and strongest muscle tendon complex in the human body (Doral et al., 2010). The AT plays an important role in storing and releasing elastic strain energy, allowing for efficient functioning of the gastrocnemius–AT complex in walking, running, and jumping (Obst et al., 2016). AT and gastrocnemius injuries are common musculoskeletal disorders, especially the former (Edama et al., 2015b; Lagas et al., 2020). Additionally, owing to increasing mechanical demands on the AT, approximately 82% of such injuries occur in sports and recreational activities (Huttunen et al., 2014; Lantto et al., 2015). Stiffness is one of the mechanical property parameters of muscle and tendon, representing the resistance of soft tissue to deformation (De Zordo et al., 2010). Muscle and tendon stiffness could objectively indicate tissue conditions such as pain and fatigue (Lichtwark et al., 2007). Stiffness measurement is a potential diagnostic method for assessing Achilles tendinopathy and AT degeneration (Obst et al., 2018). It has been reported that stiffness in Achilles tendinopathy is lower than normal AT stiffness (Coombes et al., 2018; Obst et al., 2018).

In addition to association with tendon pathology, stiffness has been widely used to represent modulations in the mechanical properties of tendons after training (Fouré et al., 2010; Siu et al., 2016). The mechanical properties of muscle and tendon can affect human daily locomotion and stability performance (Lichtwark et al., 2007). The stiffer muscle and tendon are beneficial for fast stretch-shortening cycle activities and actions involving high movement velocity (Brughelli and Cronin, 2008). It was reported that the muscle and tendon stiffness is related to the athletic performances and the economy of running (Fletcher et al., 2010; Albracht and Arampatzis, 2013). According to previous studies, the tendon is highly sensitive to mechanical loading (Arampatzis et al., 2010). Although some studies have explored the effect of exercise and training on the mechanical, material, and morphological properties of tendon and muscle, in terms of the mechanical properties of muscle and tendon, the results of these studies are inconsistent (Kubo et al., 2007, 2012; Wiesinger et al., 2015; Siu et al., 2016; Leung et al., 2017; Geremia et al., 2018). AT stiffness increased by 51–82% after 4–12 weeks of high-load plantar flexion training (Geremia et al., 2018). Leung et al. (2017) reported a more drastic increase in MG and LG stiffness after one eccentric heel drop exercise. Similarly, Siu et al. (2016) detected greater AT stiffness in the non-dominant side in frequent weight-bearing exercisers than in infrequent exercisers. In contrast, Kubo et al. (2007, 2012) found that AT stiffness was unchanged after 12 weeks of plyometric training and 8 weeks of isometric plantar flexion training, respectively. Therefore, it is necessary to understand AT and gastrocnemius stiffness variations in response to mechanical loading to improve our understanding of muscle and tendon adaptations and to provide a reference for accurate diagnosis of abnormalities.

Many clinical studies have used the healthy side as a reference to quantify changes in an affected leg when examining stiffness changes in the treatment of AT rupture or Achilles tendinopathy (McNair et al., 2013; Bohm et al., 2015). Furthermore, according to assumptions of symmetrical tendon stiffness, a lot of studies have selected only one leg to investigate differences in mechanical properties across populations (Kongsgaard et al., 2007; Stenroth et al., 2012). However, two legs show different loading during walking, even though it appears to be symmetrical (Riskowski et al., 2012; Polk et al., 2017). Even in daily life, foot dominance also could affect the symmetry of tendon properties in the lower limbs (Arampatzis et al., 2010). Moreover, there is no definitive evidence for the assumptions of symmetrical tendon properties between the legs (Bohm et al., 2014). Inconsistent with the assumption of symmetrical tendon stiffness, Bayliss et al. (2016) found that AT stiffness in preferred jumping legs was higher than in non-preferred legs among collegiate-level jumping athletes. So, one of our objectives was to determine whether AT and gastrocnemius stiffness were different between the dominant and non-dominant legs. As far as the gastrocnemius–AT complex is concerned, there is a strong correlation between the AT and gastrocnemius. However, there are differences between MG and LG in muscle structure, function, and force-generating capacity (Edama et al., 2015a). To the best of our knowledge, current research has largely focused on mixed or group muscles, with few studies examining the individual triceps calf muscles. The exact correlation between the AT and gastrocnemius, therefore, awaits further investigation.

Recently, myotonometry, a non-operator-dependent technology, has been used to quantify the mechanical properties of muscle and tendon conveniently and quickly. Similar to Young’s modulus of muscle and tendon measured by shear wave elastography, the stiffness of muscle and tendon assessed by MyotonPRO can reflect the relative stiffness properties of the soft tissue, although they are not equivalent to the true modulus of elasticity obtained from biomechanical testing *in vitro* (Kelly et al., 2018). Compared with shear wave elastography, MyotonPRO requires less experience from the operator and is less costly (Feng et al., 2018; Liu et al., 2018). However, only a few studies have reported myotonometry assessment in the gastrocnemius and AT for the athletic population (Cristi-Sánchez et al., 2019).

The purposes of this study were to (1) investigate the effects of mechanical loading on MG, LG, and AT stiffness by comparing amateur basketball players with general non-athletic subjects; (2) examine differences of MG, LG, and AT stiffness between dominant and non-dominant sides; and (3) evaluate stiffness correlations between MG, LG, and AT in basketball players and non-athletes.

## Materials and Methods

### Participants

Forty healthy male participants (aged 18–35 years) were recruited for the present study. They comprised 20 amateur basketball players and 20 general non-athletic subjects. The sample size was calculated based on a previous study (Dirrichs et al., 2019), in which the effect size between athletes and non-athletic control groups on AT stiffness was 0.92. Assuming that α at 5% and statistical power at 80%, the estimated sample size was 16 subjects per group. Therefore, 20 subjects in each group were sufficient for this experiment.

The inclusion criteria of the general non-athletic subjects were as follows: (1) ages varied between 18 and 35 years; (2) body mass index (BMI) ranged from 16 to 28 kg/m^2^; (3) no history of lower limb trauma; (4) no neurological disorders; (5) no musculoskeletal system diseases; (6) no regular exercise; and (7) participants provided written informed consent and were willing to cooperate with the researchers. Additional inclusion criteria for the basketball players were a regular history of training (at least 5 years and 6 h per week). Exclusion criteria included pain in the gastrocnemius or plantar heel or AT, plantar fasciitis and Achilles tendinopathy, metabolic and inflammatory diseases, a history of hormone therapy, treatment with corticosteroids, skin lesions above measuring sites, performed exercise within 48 h of testing, and inability to complete the full experiment.

### Equipment

The MyotonPRO (Myoton AS, Tallinn, Estonia) was used to quantify stiffness in this study. The device’s basic principles are as follows: after pre-compressing the tissue, mechanical impulses from the probe cause oscillations from the measurement of soft tissue. The MyotonPRO recorded the information and calculated the soft tissue’s mechanical parameters (Schneider et al., 2015). Among these parameters is stiffness (Newton/meter; N/m). The larger the value is, the stiffer the tissue.

### Experimental Procedures

Subjects’ exercise history was recorded based on their past and present physical activities. Demographic characteristics (age, weight, and height) were also recorded. We measured the stiffness of the dominant and non-dominant legs. The dominant leg of each subject was identified by which one they used when asked to kick a ball (Lenskjold et al., 2015; Zhang et al., 2015).

According to previous studies, the measurement region for AT stiffness was 4 cm above the calcaneal tuberosity, where Achilles tendinopathy is more likely to occur (Stenroth et al., 2012). The MG measurement site was located at 30% of the length between the popliteal fossa and lateral malleolus (Hirata et al., 2017). The LG measurement site was defined as one-third of the length between the small head of the fibula and the heel (Masood et al., 2014).

Participants were asked to rest for 5 min before stiffness measurement. For the examination, each subject adopted a prone position, with hip and knee joint fully extended, and the feet hanging over the edge of the examination couch. The subjects were asked to completely relax for the whole examination. Measurement regions were marked by the same experienced physical therapist. LG, MG, and AT stiffness were obtained at neutral position (ankle joint dorsiflexion 0°) and 10° dorsiflexion (10° DF) of the ankle joint (Taş and Salkın, 2019). Three measurements were taken at each measurement region and the mean values were used for statistical analysis. The angle of the ankle joint was maintained by a customized and movable ankle–foot orthosis. The order of measurements was LG, MG, and AT. The subjects relaxed for 5 min after completing measurement at ankle neutral position. Then, MG, LG, and AT stiffness were measured at ankle dorsiflexed 10°. During the stiffness measurement, the MyotonPRO’s probe is placed on and perpendicular to the surface of the measurement region (Sohirad et al., 2017).

### Statistical Analyses

All statistical analyses were performed using SPSS software (SPSS version 22.0, IBM, United States). The sample size calculations were performed using the G^∗^Power program (G^∗^Power 3.1.9). Descriptive data and all stiffness data were represented as mean ± standard deviation. The normality of all data distribution was assessed using the Shapiro–Wilk test. Homogeneity of variances was tested using Levene’s test. BMI was calculated by the following formula: BMI = weight (kg)/height (m^2^). Comparisons between stiffness in the healthy subjects and the amateur basketball players were evaluated using an independent sample *t* test. A paired-sample *t* test was performed for stiffness differences on the bilateral sides of participants. Pearson correlation analysis (*r*) was used to analyze the correlation between AT, LG, and MG stiffness. A histogram was generated using GraphPad Prism 8.

## Results

### Demographic Information

Subjects’ demographic characteristics and duration of training are presented in Table 1. No differences were observed between the two groups in terms of basic characteristics, such as age, weight, height, and BMI.

**TABLE 1:** The characteristics of the subjects.

	Amateur basketball players (*M* ± SD)	General non-athletic subjects (*M* ± SD)
Age (years)	30.30 ± 2.70	29.8 ± 3.10
Height (m)	1.77 ± 0.04	1.76 ± 0.05
Weight (kg)	73.80 ± 6.27	74.35 ± 7.53
BMI (kg/m^2^)	23.50 ± 1.59	24.12 ± 1.68
Training years	14.81 ± 4.10	
Training hours/week	7.72 ± 1.20	

### MG, LG, and AT Stiffness Between the Amateur Basketball Players and the General Non-athletic Subjects

**FIGURE 1:**
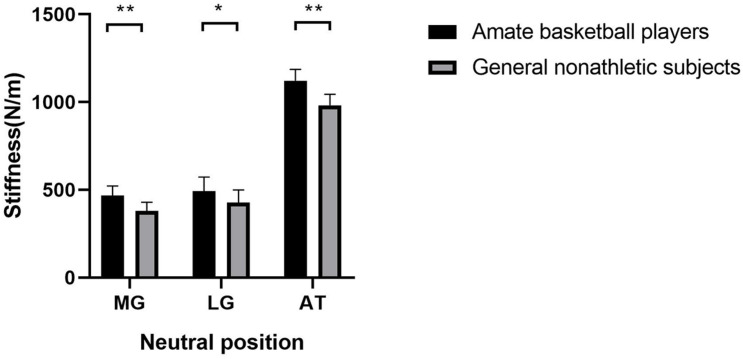
The mean stiffness of the lateral gastrocnemius (LG), medial gastrocnemius (MG), and Achilles tendon (AT) between amateur basketball players and general non-athletic subjects at ankle neutral position. Amateur basketball players (black) and general non-athletic subjects (gray). **P < 0.001, *P < 0.05.

**FIGURE 2:**
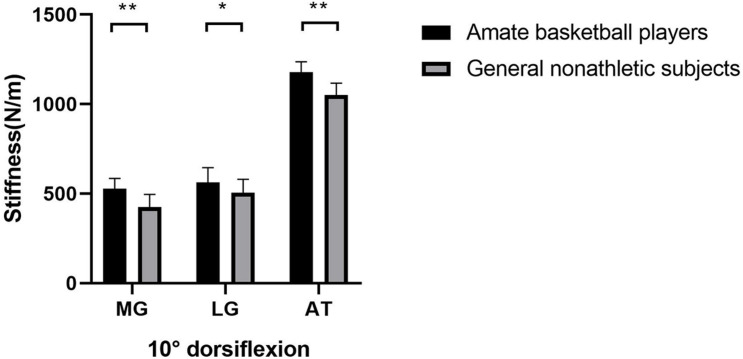
The mean stiffness of the LG, MG, and AT between amateur basketball players and the general non-athletic subjects at dorsiflexion 10° of ankle joint. Amateur basketball players (black) and general non-athletic subjects (gray). **P < 0.001, *P < 0.05.

As shown in Figure 1, the gastrocnemius and AT stiffness of amateur basketball players was greater than that of the general non-athletic subjects in the ankle neutral position (amateur basketball players vs. general non-athletic subjects; MG: 468.00 ± 53.31 vs. 379.55 ± 50.27; LG: 491.90 ± 80.89 vs. 426.40 ± 73.03; and AT: 1,122.35 ± 65.04 vs. 982.10 ± 61.98). Similar results were observed in dorsiflexion 10° of ankle joint (amateur basketball players vs. general non-athletic subjects). MG stiffness in basketball players was greater than that in non-athletes (528.25 ± 57.21 vs. 424.70 ± 70.58). LG stiffness was greater in basketball players (562.45 ± 83.07 vs. 504.75 ± 76.26). AT stiffness was greater in basketball players than that in non-athletic subjects (1,179.00 ± 57.59 vs. 1,050.95 ± 66.31; Figure 2).

### MG, LG, and AT Stiffness Between the Non-dominant and Dominant Sides

Tables 2, 3 reveal the stiffness of MG, LG, and AT at ankle neutral position and ankle dorsiflexion 10° in amateur basketball players and general non-athletic subjects, respectively. There was no difference in MG, LG, and AT stiffness between the non-dominant and dominant sides (*P* > 0.05).

**TABLE 2:** The stiffness of the medial gastrocnemius (MG), lateral gastrocnemius (LG), and Achilles tendon (AT) in amateur basketball players.

Amateur basketball players		Dominant side (*M* ± SD)	Non-dominant side (*M* ± SD)	*P*
Neutral position	MG	468.00 ± 53.31	445.90 ± 76.34	0.31
	LG	491.90 ± 80.89	516.50 ± 69.63	0.24
	AT	1,122.35 ± 65.04	1,092.40 ± 70.68	0.14
Dorsiflexion 10°	MG	528.25 ± 57.21	497.70 ± 97.93	0.23
	LG	562.45 ± 83.07	592.30 ± 80.49	0.09
	AT	1,179.00 ± 57.59	1,138.05 ± 88.80	0.13

**TABLE 3:** The stiffness of the MG, LG, and AT in the general non-athletic subjects.

General non-athletic		Dominant side	Non-dominant side	*P*
subjects		(*M* ± SD)	(*M* ± SD)	
Neutral position	MG	379.55 ± 50.27	395.40 ± 57.92	0.23
	LG	426.40 ± 73.03	440.40 ± 59.72	0.43
	AT	982.10 ± 61.98	990.60 ± 62.67	0.67
Dorsiflexion 10°	MG	424.70 ± 70.58	450.85 ± 97.11	0.13
	LG	504.75 ± 76.26	520.00 ± 70.09	0.35
	AT	1,050.95 ± 66.31	1,069.00 ± 87.64	0.49

### The Relationship Between Stiffness of the AT and MG and LG

Further analysis of the data reveals a significant correlation between the AT and MG in amateur basketball players (*r* = 0.726 and *P* = 0.001 at the neutral position, *r* = 0.687 and *P* = 0.001 at the ankle dorsiflexed position). For the basketball players, no significant correlation was apparent in stiffness results between the AT and LG, regardless of ankle position (*P* > 0.05). Table 4 shows the *r* and *P* values of the AT and MG and LG for different ankle joint positions in amateur basketball players and general non-athletic subjects.

**TABLE 4:** The relationship of the stiffness between the AT and MG and LG.

		AT (*r*)	
		Amateur basketball players	General non-athletic subjects
Neutral position	MG	0.726/0.001**	0.349/0.132
	LG	0.389/0.090	0.312/0.180
10° dorsiflexion	MG	0.687/0.001**	0.260/0.268
	LG	0.244/0.299	0.125/0.599

**P ≤ 0.001; *r*, correlation coefficient.

## Discussion

This present study evaluated the stiffness of the gastrocnemius–AT complex between the non-dominant and dominant sides in amateur basketball players and general non-athletic subjects. We found a greater stiffness in the basketball players than the non-athletes. No significant difference was investigated between the non-dominant and dominant sides. Additionally, a significant positive correlation was obtained between AT and MG stiffness in amateur basketball players.

### MG, LG, and AT Stiffness Between the Amateur Basketball Players and General Non-athletic Subjects

This study investigated the effects of chronic loading on the mechanical properties of tendons and muscles using amateur basketball players as examples. We found that AT and gastrocnemius stiffness was greater in basketball players than that in the general non-athletic subjects. Variation of tendon mechanical properties is one of the proposed mechanisms for loading adaptation (Kongsgaard et al., 2007; Arampatzis et al., 2010). A stiffer tendon was detected in athletes who had been training for many years, suggesting that it was adapted to a long-term exercise program (Arampatzis et al., 2010). Many studies have explored the effects of habitual loading on the mechanical properties of AT and the gastrocnemius. Our results are consistent with previous studies that found that the AT became stiffer after chronic mechanical loading such as exercise (Albracht and Arampatzis, 2013; Siu et al., 2016; Dirrichs et al., 2019). Siu et al. (2016) demonstrated that frequent weight-bearing exercisers had greater AT stiffness on the non-dominant side than did infrequent exercisers. Healthy semiprofessional running athletes exhibit a significantly higher value of AT stiffness than the non-athletic general population on both the left and right sides (Dirrichs et al., 2019). Some 14 weeks of resistance training intervention resulted in a 16% increase in triceps surae aponeurosis stiffness (Albracht and Arampatzis, 2013). However, inconsistent with our results, 20 healthy participants were recruited to measure the elastic modulus of the calf muscles using shear wave elastography at the beginning and end of a 30-min running task, and they found no significant change in the LG and MG after the exercise (Ohya et al., 2017). In that study, the participants were asked to perform a 30-min running task. In our study, the subjects underwent long-term basketball player exercises. The mechanical loading level has been proven to determine muscle and tendon adaptation (Bohm et al., 2014). Besides, exercise types and subjects’ training intensity also have an impact on muscle and tendon adaptation (Kubo et al., 2007).

One study indicated an increased type I collagen in the AT’s peritendinous tissue after performing physical training (Langberg et al., 2001; Bohm et al., 2014). This is one of the mechanisms to explain increased tendon stiffness. In addition, variations in collagen fibril morphology and collagen molecular cross-linking levels can also cause tendon adaptation changes (Kjaer et al., 2009). Muscle and tendon mechanical properties have been shown to affect sports performance (Kubo et al., 2015). From a biomechanical perspective, stiffer muscles use tendon elasticity more efficiently (Muraoka et al., 2005; Obst et al., 2016). Kubo et al. (2015) investigated the relationship between stiffness of plantar flexors and running performance in long-distance runners. They found a positive relationship between the stiffness of the plantar flexors and the best official record in a 5,000-m race. A stiffer vastus lateralis can be beneficial to athletic performance in both sprinters and long-distance runners (Miyamoto et al., 2019). AT stiffness has been confirmed to correlate with muscle strength in the triceps surae (Epro et al., 2018). Also, modifications in tendon stiffness can meet increased functional demands due to changes in muscle force (Raiteri et al., 2018). The gastrocnemius–AT complex plays a critical role in the sport-specific demands of basketball (Lemme et al., 2019). Therefore, the stiffer AT and gastrocnemius of amateur basketball players may contribute to improving their performance on the court. However, in addition to physiological adaptive muscle and tendon change, excessive mechanical loading can lead to tendinopathy and tendon degeneration (Grimaldi et al., 2015).

Minimal detectable change (MDC) could provide a value to reflect a true change as a reference for further study. In our previous study, we found an excellent intra- (ICC = 0.85–0.94) and inter-rater reliability (ICC = 0.87–0.92) for evaluating AT stiffness using the MyotonPRO, with a relatively low MDC (MDC less than 45 N/m; Liu et al., 2018). In terms of this finding, measurements of the AT stiffness should be greater than 45 N/m to reflect real change. Also, the differences in means stiffness values between the amateur basketball players and the general non-athletic subjects exceeded the value of MDC in the present study, suggesting that the difference in measurements is a “real” difference. Regrettably, we did not obtain the MDC of gastrocnemius stiffness measurements.

### MG, LG, and AT Stiffness Between the Non-dominant and Dominant Sides

Our study demonstrates symmetric stiffness in the AT and gastrocnemius between the dominant and non-dominant lower limbs, and this result provides evidence for the assumptions of symmetrical tendon properties between the legs. In future studies, the stiffness of the healthy side could be measured as a reference to quantify degenerative or pathological changes of the affected leg. The results of our study were consistent with various other research findings. A study of moderately active individuals examined similar AT stiffness between non-dominant and dominant legs (Bohm et al., 2015). Another study investigated muscle structure and stiffness in lower limbs and observed no difference in MG and LG stiffness and muscle thickness between dominant and non-dominant lower limbs in professional badminton players (Bravo-Sánchez et al., 2019). Similar results were also observed in the AT stiffness of semiprofessional running athletes and the non-athletic general population (Dirrichs et al., 2019). Cristi-Sánchez et al. (2019) assessed AT stiffness between the dominant and the non-dominant limbs in elite soccer players using the MyotonPRO. Just like us, they located the measurement site 4 cm above calcaneal tuberosity, and they reported stiffness of 1,075.0 ± 100.8 and 1,031.0 ± 115.9 N/m in the dominant and non-dominant limbs. This is similar to our AT stiffness results for amateur basketball players (dominant limbs: 1,122.35 ± 65.04 N/m; non-dominant limbs: 1,092.40 ± 70.67 N/m). The small differences in AT stiffness maybe because they did not hold the ankle joint angle in the neutral position using the ankle–foot orthosis. However, conflicting results were observed by a study that found the AT stiffness in preferred jumping legs was higher than for non-preferred jumping legs in collegiate-level jumping athletes (Bayliss et al., 2016). Couppé et al. (2008) found higher patellar tendon stiffness in the lead extremity than non-lead extremity in elite fencers and badminton players. Moreover, they also concluded that the change in mechanical properties was primarily the result of a change in tendon size. As stated before, the studied subjects, the stiffness measurement region, and their sports are different, which may account for discrepancies in the findings. According to one report (Kubo et al., 2012), AT stiffness did not increase significantly within 2 months of isometric plantar flexion training, but became statistically significant after 3 months of training. A study proposed that strain magnitude applied to AT has a threshold, and that when this is exceeded, tendon stiffness will change (Arampatzis et al., 2007). Although lower limb asymmetries during typical stop-jump movement potentially lead to overloading of dominant limbs in jumping sports (Edwards et al., 2012), this limb dominance may not provide enough differential stimulus to induce asymmetric adaptation (Benítez-Martínez et al., 2019). Symmetrical stiffness may also be due to a similar amount of stimulus of both lower limbs after years of training and matches.

### The Relationship Between the Stiffness of the AT and MG and LG

In this study, we observed a significant positive correlation between the stiffness of AT and MG in amateur basketball players. Many studies have suggested architectural and functional differences between the MG and LG (Albracht et al., 2008; Toumi et al., 2016). Toumi et al. (2016) proposed that the muscle volume of MG was higher than that of LG in the gastrocnemius–AT complex. Although MG and LG have the same effect on ankle plantar flexion, they contribute to different degrees. MG provides more than 70% of muscle strength, about twice as much as LG does (Albracht et al., 2008). The fascicles of the tendons of LG and soleus muscles comprise the deep part of AT, while the MG forms the superficial part of the AT (Edama et al., 2015a). The imbalance between MG and LG can contribute to the development of Achilles tendinopathy or AT pain (Mogi et al., 2018). Structural or functional differences between the LG and MG may influence their correlation with the AT.

Many studies reported that the AT’s mechanical properties were closely related to the MG in different exercise programs. Hirata et al. (2017) reported a significant decrease in MG stiffness and no significant change in LG after static stretching. Riemann et al. (2011) demonstrated that MG activation was significantly greater than LG during the eccentric phase of heel-raise exercise. Crill et al. (2014) observed that GM fascicle length increased 12% in patients with Achilles tendinopathy after 6 weeks of eccentric training, but there was no significant change in the LG. This is the first study to investigate the relationship between the AT and gastrocnemius stiffness using the MyotonPRO. The findings of the present study suggest a closer correlation between MG and AT in amateur basketball players. This finding may provide a new treatment idea for AT disorders. Reducing MG tension may be considered to be one effective treatment for AT injury prevention and rehabilitation. Since only healthy participants were recruited in this study, more research is needed to investigate the correlation between the MG and AT in patients with AT disorders and to explore whether current management programs for AT disorders need to be adapted for MG.

### Limitations

We should mention limitations to the present study. First, only one muscle and tendon point was evaluated, which cannot represent stiffness variations in other parts of the complex. Although we suggested that participants be completely relaxed throughout the experiment, this is difficult to control precisely. Considering that the stiffness is related to the degree of muscle contraction, as a result, the stiffness values could have been overestimated in this study. In addition, we evaluated only variations in mechanical properties of the gastrocnemius–AT complex, but did not assess changes in its morphological properties. Finally, we investigated the mechanical properties of the gastrocnemius–AT complex only in healthy subjects. Therefore, a subsequent experiment should focus on the stiffness of Achilles tendinopathy under excessive habitual loading.

## Conclusion

The stiffness of the gastrocnemius and AT in basketball players is significantly greater than that in the general non-athletic subjects. This is possibly caused by repeated training effects. The symmetric stiffness of the AT and gastrocnemius exists both in amateur basketball players and in generally healthy subjects. The present study suggests a significant correlation between the MG stiffness and AT stiffness in amateur basketball players.

## Data Availability Statement

The raw data supporting the conclusions of this article will be made available by the authors, without undue reservation.

## Ethics Statement

The studies involving human participants were reviewed and approved by the Human Subjects Ethics Committee of Luoyang Orthopedic Hospital of Henan Province. The patients/participants provided their written informed consent to participate in this study.

## Author Contributions

T-TC and ZL performed all the experiments. Z-JZ designed the experiments. Z-JZ and X-QW analyzed the experimental results. T-TC and ZL wrote the manuscript. All authors reviewed and approved the submission of the manuscript.

## Conflict of Interest

The authors declare that the research was conducted in the absence of any commercial or financial relationships that could be construed as a potential conflict of interest.
